# Nanoindentation of GaSe thin films

**DOI:** 10.1186/1556-276X-7-403

**Published:** 2012-07-17

**Authors:** Sheng-Rui Jian, Shin-An Ku, Chih-Wei Luo, Jenh-Yih Juang

**Affiliations:** 1 Department of Materials Science and Engineering, I-Shou University, Main Campus No.1, Sec. 1, Syuecheng Rd., Dashu District, Kaohsiung, 84001, Taiwan; 2 Department of Electrophysics, National Chiao Tung University, Hsinchu, 300, Taiwan

**Keywords:** GaSe thin films, XRD, Nanoindentation, Hardness

## Abstract

The structural and nanomechanical properties of GaSe thin films were investigated by means of X-ray diffraction (XRD) and nanoindentation techniques. The GaSe thin films were deposited on Si(111) substrates by pulsed laser deposition. XRD patterns reveal only the pure (000 *l*)-oriented reflections originating from the hexagonal GaSe phase and no trace of any impurity or additional phases. Nanoindentation results exhibit discontinuities (so-called multiple ‘pop-in’ events) in the loading segments of the load–displacement curves, and the continuous stiffness measurements indicate that the hardness and Young’s modulus of the hexagonal GaSe films are 1.8 ± 0.2 and 65.8 ± 5.6 GPa, respectively.

## Background

The most unique structural feature of the family of III-VI semiconductors is the existence of layers within which the atomic bonds are mainly covalent with a certain degree of ionic component. GaSe is a layered III-VI chalcogenide semiconductor with a number of interesting properties for electrical and nonlinear optics applications, such as compound semiconductor heterostructure devices [1], IR detectors [2], and solar cells [3]. However, while most of the previous researches have been concentrated on its electrical and optical characteristics for device applications, researches on the mechanical properties have not drawn equal attention. A quadruple layer of GaSe building block consists of two Ga and two Se sub-layers with the stacking sequence of Se-Ga-Ga-Se, where the Se-Ga and Ga-Ga bonds are covalent within the quadruple layers and the Se-Se bond between adjacent quadruple layers is due to van der Waal forces [4]. As a result, significant differences as compared to most III-V and II-VI semiconductors are expected and accurate measurements of the mechanical properties of GaSe thin films are required, since such parameters are critical for making structural elements as well as functional devices.

Due to its high sensitivity, excellent resolution, and easy operation, nanoindentation has been widely used for characterizing the mechanical properties of various nanoscale materials [5,6] and thin films [7-9]. Among the mechanical properties of interest, hardness, Young’s modulus, and elastic/plastic deformation behaviors can be readily obtained from nanoindentation measurements. Through the analyses on the load–displacement curves obtained during the nanoindentation, the hardness and Young’s modulus of the test material can be easily obtained following the methods proposed by Oliver and Pharr [10]. However, in the case of thin films, the responses after certain penetration depth may arise from the film and underneath substrate, and the obtained mechanical properties may not be completely reflecting the intrinsic properties of the films. Thus, indentation with contact depths of less than 10 % of the films’ thickness is needed to obtain intrinsic film properties and avoid the influence of the substrate [11]. In addition, it is very difficult to obtain meaningful analytical results for indentation depths less than 10 nm because of the equipment limitations. Hence, it is not possible to obtain substrate-independent results for films thinner than 100 nm. In order to get an insight on the influence of the substrate and obtain the intrinsic properties for films less than 100 nm thick, it is essential to monitor the mechanical properties as a function of depth. In this work, we used a dynamic approach, referred as continuous stiffness measurement (CSM) mode [12], to continuously monitor the hardness and Young’s modulus values as a function of the indentation depth. Herein, in this study, Berkovich nanoindentation-induced multiple pop-in behaviors were observed and the mechanical properties of the hexagonal GaSe thin films were obtained by analyzing the nanoindentation load–displacement curves.

## Methods

GaSe thin films were deposited on the 0.3-mm-thick Si(111) substrates at 475 °C by pulsed laser deposition (PLD). The target used was a GaSe single crystal grown by vertical Bridgman method. The thickness of GaSe films was about 200 nm. The structural features of the obtained GaSe films were inspected by X-ray diffraction (XRD; Bruker D8 Advance TXS with Cu-Kα radiation, *λ* = 1.5406 Å, Madison, WI, USA). The mechanical properties (hardness and Young’s modulus) of GaSe thin films were investigated using an MTS Nano Indenter® XP instrument with a three-sided pyramidal Berkovich indenter tip (Eden Prairie, MN, USA). Prior to applying loads to the GaSe films, indentations were conducted on the standard sample (fused silica with a Young’s modulus of 68 to 72 GPa) to obtain the reasonable loading range. The CSMs [12] were carried out by superimposing small-amplitude, 75-Hz oscillations on the force signal to record stiffness data along with load and displacement data dynamically. Firstly, the indenter was loaded and unloaded three times to ensure that the tip was properly in contact with the surface of the materials and that any parasitic phenomenon is released from the measurements. Then, the indenter was loaded for a fourth and final time at a strain rate of 0.05/s, with a 60-s holding period inserted at peak load in order to avoid the influence of creep on unloading characteristics, which were used to compute the mechanical properties of the specimen. In addition, for the sake of obtaining steady mechanical characteristics and preventing interference from environmental fluctuation factor, each test was performed when the thermal drift dropped down to 0.01 nm/s. The analytic method developed by Oliver and Pharr [10] was used to determine the hardness (*H*) and Young’s modulus (*E*) of the GaSe thin film from the load–displacement curves.

**Figure 1:**
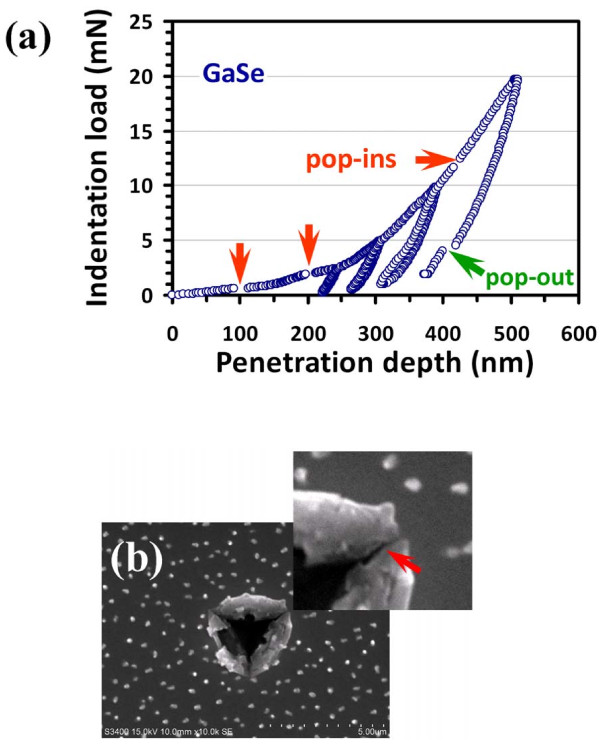
**Cyclic load–displacement curves and SEM micrograph on an ‘indented’ GaSe thin film.** (**a**) The cyclic load–displacement curves obtained at an indentation load of 20 mN for the GaSe films. Notice that both multiple ‘pop-in’ and ‘pop-out’ phenomena are observable in the load–displacement curves. (**b**) SEM micrograph of an ‘indented’ GaSe thin film showing the cracking (see the red arrow) along the corners and ‘pile-ups’ along the edges of the Berkovich indentation after an indentation load of 20 mN.

Moreover, in order to reveal the fracture behavior of GaSe thin films, cyclic nanoindentation tests were also performed in this study. These tests were carried out by the following sequences: First, the indenter was loaded to some chosen load and then unloaded by 90 % of the previous load, which completed the first cycle. It was then reloaded to a larger chosen load and unloaded by 90 % for the second cycle. Figure 1a illustrates a typical cyclic indentation test repeated for four cycles, revealing features such as pop-ins (discontinuities in the loading segment of the load–displacement curve) and even pop-out in the unloading segment of higher load tests. More detailed discussion on these features will be given later. It is noted that in each cycle, the indenter was held for 10 s at 10 % of its previous maximum load for thermal drift correction and for assuring that complete unloading was achieved. The thermal drift was kept below ±0.05 nm/s for all indentations considered in this study. The same loading/unloading rate of 10 mN/s was used. After that, scanning electron microscopy (SEM) studies were performed with Hitachi S3400N (Chiyoda-ku, Japan) at 7-kV operating voltage in secondary electron mode.

## Results and discussion

**Figure 2:**
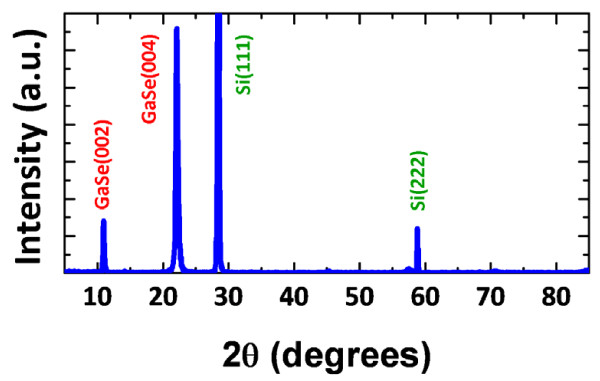
**XRD spectrum of GaSe thin films, showing the purely (000** ***l*)-oriented features of the hexagonal-phase GaSe.**

Figure 2 shows the XRD *θ*-2*θ* pattern for GaSe thin films grown on (111)-oriented Si substrates. It is evident that only the (000 *l*) diffraction peaks of the GaSe thin film are observed. The obtained values of *d*-spacing are in good agreement with the previous study [13], confirming that the present films are indeed purely *c*-axis-oriented hexagonal-structured GaSe.

**Figure 3:**
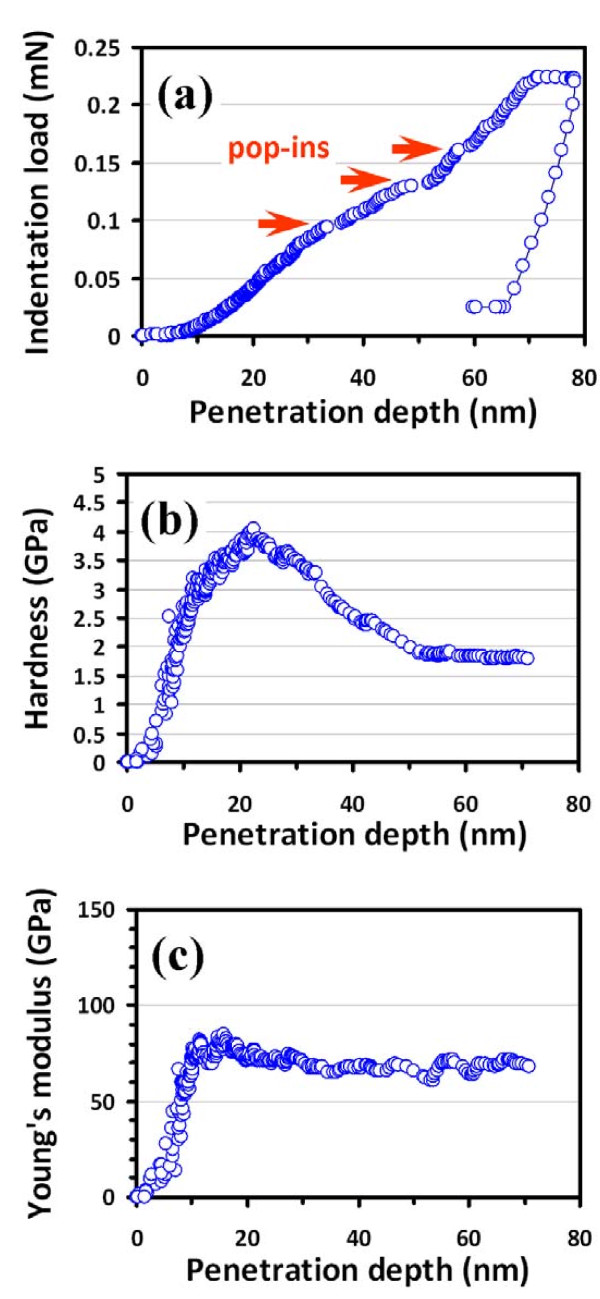
**Nanoindentation test results.** (**a**) A load–displacement curve of the GaSe thin film showing the multiple pop-ins during loading, (**b**) hardness-displacement curve, and (**c**) Young's modulus-displacement curve for the GaSe thin film.

Figure 3a shows the typical nanoindentation load–displacement curve obtained for hexagonal GaSe films. The total penetration depth into the GaSe thin film was approximately 80 nm with a peak load of approximately 0.23 mN, which is well within the nanoindentation criterion suggested by Li et al. [14], which states that the nanoindentation depth should never exceed 30 % of the films’ thickness or the size of nanostructures under test. The results displayed in Figure 3, thus, should reflect primarily the intrinsic properties of the present GaSe thin films. It is evident from Figure 3a that there are several pop-in events occurring along the loading segment of the load–displacement curve with the threshold loading of the first pop-in being around 0.1 mN. The plastic deformation behavior observed here is consistent with those reported for InSe and GaSe single crystals by Mosca et al. [15], albeit that in single crystal cases, the threshold of pop-in event occurred at slightly higher loadings (approximately 0.3 mN). Moreover, we note that the threshold loading of the present GaSe films is much lower than that of other hexagonal III-V semiconductors, such as AlN films deposited on *c*-plane sapphire substrates where the first pop-in occurred at approximately 0.2 mN [16]. As pointed out by Mosca et al. [15], the plastic deformation of the layer-structured InSe and GaSe may involve breaking of In-In (or Ga-Ga) bonds, activation of dislocation slip, twinning, and bending mechanisms. On the other hand, as can be seen in both Figures 1a and 3a, the deformation between pop-ins is predominantly elastic even with a load up to 20 mN, suggesting that the slip process should play a prominent role in the deformation mechanisms of this layered material. Furthermore, since the pop-ins are generally closely related to the sudden collective activities of dislocations (such as dislocation generation or movement bursts), it is suggestive that during the course of plastic flow, preferential collective slips might be occurring by activating the pre-existing dislocations during thin film growth or following nucleation of dislocations when some critical strain has reached [15].

With the CSM measurements, the penetration depth-dependent hardness and Young’s modulus of the hexagonal GaSe thin film were calculated directly from the load–displacement data following the analytic method developed by Oliver and Pharr [10]. The results are displayed in Figure 3b,c, respectively. In Figure 3b, the penetration depth-dependent hardness of GaSe thin films can be roughly divided into two stages, namely the initial increase to a maximum value and an asymptotic decrease toward a nearly constant value after the penetration depth reaches 50 nm. The increase in hardness at small penetration depth is usually attributed to the transition between purely elastic to elastic/plastic contact whereby the hardness is really reflecting the mean contact pressure and is not representing the intrinsic hardness of GaSe. Only under a condition of a fully developed plastic zone does the mean contact pressure represent the hardness. When there is no plastic zone, or a partially formed plastic zone, the mean contact pressure (which is measured using the Oliver and Pharr method) is less than the nominal hardness. After the first stage, the hardness gradually decreases with the increasing penetration depth and eventually reaches a more or less constant value of 1.8 ± 0.2 GPa, which is in good agreement with that of the single crystal GaSe (2.0 ± 0.4 GPa) [15]. It is also noted that within the penetration depth of 50 nm, the hardness appears to be quite ‘noisy’ as a function of the penetration depth, presumably due to the extensive dislocation activities being activated in this stress range.

Figure 3c shows the plot of Young’s modulus as a function of penetration depth for the GaSe thin film derived using the method of Oliver and Pharr [10]. Unlike that displayed in Figure 3b for the hardness, the Young's modulus appears to be relatively insensitive to the penetration depth of the Berkovich indenter after the first 20 nm, within which the changes have been attributed to the transition between purely elastic to elastic/plastic contact. Moreover, the obtained Young's modulus for the GaSe films is 65.8 ± 5.6 GPa, which is much larger than the value of 33 ± 3 GPa reported for GaSe single crystals [15]. Mechanical properties of materials are size-dependent due to the influences of surface stress effect [17,18]. However, it is not clear at present why the Young's modulus exhibits such a substantial difference between the thin films and single crystal of GaSe, while the hardness of both remains almost the same.

Now turning back to Figure 1a, the cyclic load–displacement curves obtained with the Berkovich indenter for the hexagonal GaSe thin film clearly displays multiple pop-ins in the indentation loading curves. Although at relatively lower applied loadings (<2 mN) the behaviors are similar to that displayed in Figure 3a, at higher applied loadings, especially the last segment, it may have arisen from a very different deformation mechanism. In particular, due to the large indentation depth, the pop-out event observed in the last segment of the unloading curve might be related to the indentation-induced phase transition occurring in the underneath Si substrate [19]. Moreover, as revealed by the SEM photograph shown in Figure 1b, there are cracks (see the red arrow in the inset of Figure 1b) and pile-ups along the corners and edges of the residual indented pyramid, respectively. Thus, the cause of the ‘later’ multiple pop-ins may have been complicated by the involvement of the Berkovich indentation-induced cracking on the surface of the hexagonal GaSe thin films.

A closer look at the loading curves displayed in Figures 1a and 3a reveals that the multiple pop-ins do not exactly coincide at the same indenter penetration depths. Since each curve is associated with a specific stress rate depending on the maximum indentation load, it suggests that the first pop-in event is not thermally activated. Instead, these phenomena have been ubiquitously observed in a wide variety of materials and are usually attributed to dislocation nucleation or/and propagation during loading [20-22] or micro-cracking [23]. Moreover, in order to check the validness of attributing the abovementioned pop-out event exhibited in the unloading curve to the indentation-induced phase transition occurring in underneath Si substrates, we have repeated the tests many times. It was confirmed that the pop-out event occurs whenever the penetration depth exceeds 200 nm, the thickness of the present GaSe thin films. The cubic phase Si is known to exhibit pressure-induced phase transformation during nanoindentation. During the course of loading, the cubic Si will transform into metallic Si phase (Si-II) when a certain pressure is reached. While unloading, pressure release will lead to further transformation into amorphous Si or a mixture of bcc Si and rhombohedral Si phases depending on the unloading rate [19,24]. On the other hand, the pressure-induced structural phase transition in GaSe has been investigated by diamond anvil cell experiments previously [25]. The magnitude of pressure required to induce phase transitions is significantly higher than the apparent room-temperature hardness of the GaSe thin film measured in this study. Furthermore, the multiple pop-ins have been reported previously in hexagonal-structured sapphire [26], GaN thin films [27], and GaN/AlN multilayers [22], and evidences have indicated that the primary nanoindentation-induced deformation mechanism in these hexagonal-structured materials is nucleation and propagation of dislocations or crack formations. It is thus quite plausible to state that similar mechanisms must have been prevailing in the present GaSe thin films.

Within the scenario of the dislocation nucleation and propagation mechanism, the first pop-in event naturally reflects the transition from perfectly elastic to plastic deformation, that is, it is the onset of plasticity in the GaSe thin film. Under this circumstance, the corresponding critical shear stress ($\tau_{\max}$) under the Berkovich indenter at an indentation load, $P^{*}$, where the load–displacement discontinuity occurs, can be determined by using the following relation [28]:

(1) $$\tau_{\max}=0.31{\left(\frac{6P^{*}E^{2}}{\pi^{3}R^{2}}\right)}^{1/3}\text{,}$$

where *R* is the radius of the tip of the indenter. The obtained maximum shear stress, $\tau_{\max}$, for the GaSe thin film is approximately 0.6 GPa. This $\tau_{\max}$ is responsible for the homogeneous dislocation nucleation within the deformation region underneath the indenter tip. Classical dislocation theory predicts that the free energy required for homogeneous dislocation nucleation of a circular dislocation loop with radius, *r*, under the action of a uniform shear stress is given by the following relation [29]:

(2) $$F=\gamma_{\text{dis}}2\pi \;r-\tau_{\max}\;b\pi \;r^{2}\text{,}$$

and the elastic self-energy, $\gamma_{\text{dis}}$, of a fully circular dislocation loop in an infinite isotropic elastic solid is given by

(3) $$\gamma_{\text{dis}}=\frac{Gb^{2}}{8\pi }\frac{2-v_{\text{film}}}{1-v_{\text{film}}}\left[\ln\left(\frac{4r}{r_{\text{core}}}\right)-2\right]\text{,}$$

where *b* is the magnitude of the Burgers vector (approximately 0.3 nm), *G* is the shear modulus of the GaSe thin film (approximately 25 GPa), $v_{\text{film}}$ is Poisson's ratio (assumed to be approximately 0.25), and $r_{\text{core}}$ is the radius of the dislocation core. In Equation 2, the free energy has a maximum value at a critical radius, $r_{\text{c}}$, i.e., dF/dr=0 when $r=r_{c}$. Moreover, the formation free energy for a dislocation loop with size $r_{c}$ has to be in the order of the thermal energy, kT. However, since kT is very small compared to the two energy terms on the right-hand side of Equation 2, one can use $F_{c}\approx 0$ as an additional condition for calculating $r_{\text{c}}$[29], yielding

(4) $$r_{\text{c}}=\frac{2\gamma_{\text{dis}}}{b\tau_{\max}}$$

and

(5) $$r_{\text{c}}=\frac{e^{3}}{4}r_{\text{core}}\approx 5\;r_{\text{core}}\text{.}$$

Here, the values of $r_{\text{core}}\approx 0.5$ nm and $r_{\text{c}}\approx 2.3$ nm can be obtained.

**Figure 4:**
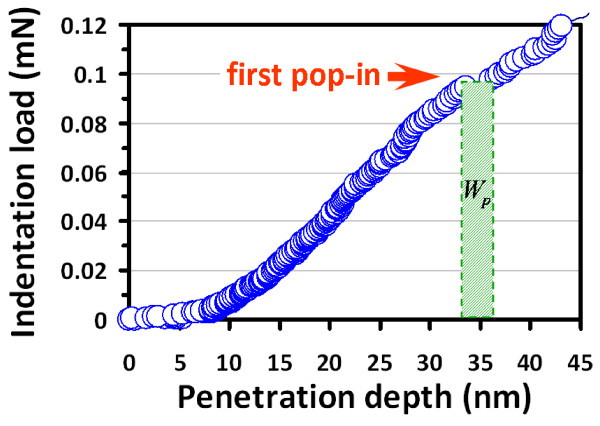
**Corresponding pop-in event.** The corresponding pop-in event (see the arrow) from Figure 3a is zoomed in, where the plastic strain work is denoted as *W*_p_ (critical indentation loading times the displacement).

The number of dislocation loops formed during the first pop-in can, thus, be estimated from the work done associated with the pop-in event. From the shaded area depicted in Figure 4, this work is estimated to be approximately $0.3\times 10^{-12}$ nm, implying that approximately $10^{5}$ dislocation loops with critical diameter might have been formed during the pop-in event. This number is low and is consistent with the scenario of homogeneous dislocation nucleation-induced pop-in, instead of activated collective motion of pre-existing dislocations [30]. When the total dissipation energy, namely the area between the loading and unloading curves shown in Figure 3a, is taken as the energy to generate dislocations with critical radius, as high as approximately $3\times 10^{6}$ dislocation loops may be formed within a loading-unloading curve. Although it is not realistic to assume that all the dissipated indentation energy was entirely transferred to generate dislocation loops, the estimation has, nevertheless, provided an upper limit for the number of dislocation loops with critical radius in the initial state.

## Conclusions

The XRD and nanoindentation techniques were used to investigate the structural features and nanomechanical properties of hexagonal GaSe thin films prepared by PLD. The main findings are briefly summarized as follows:

1. XRD analysis showed that GaSe thin films grown on Si(111) substrates are purely (000 *l*)-oriented hexagonal phase.

2. Nanoindentation results indicate that the values of hardness and Young's modulus of the hexagonal GaSe thin films are 1.8 ± 0.2 and 65.8 ± 5.6 GPa, respectively. Although the hardness value is in good agreement with those obtained from bulk single crystals (2.0 ± 0.4 GPa), the value of Young's modulus of the present films is more than twice larger than that of the bulk single crystals (33 ± 3 GPa). The reason for the apparent discrepancy is not clear at present.

3. Similar to many hexagonal-structured semiconductor materials, the primary deformation mechanism for GaSe is governed by nucleation and propagation of dislocations or crack formations. Based on the scenario, the nanoindentation-induced generation of dislocation loops associated with the first pop-in event was estimated to be in the order of approximately $10^{5}$ with a critical radius ($r_{\text{c}}\approx 2.3$ nm). The obtained dislocation density is relatively low and is consistent with the scenario of homogeneous dislocation nucleation-induced first pop-in event.

## Competing interests

The authors declare that they have no competing interests.

## Authors’ contributions

SRJ designed the project of experiments, performed the nanoindentation analysis and SEM measurements, and drafted the manuscript. SAK, CWL, and JYJ carried out the growth of GaSe thin films and performed the XRD measurements. All authors read and approved the final manuscript.

## Authors’ information

SRJ is an associate professor in the Department of Materials Science and Engineering, I-Shou University, Kaohsiung 84001, Taiwan. SAK is a Ph.D. student, CWL is an associate professor, and JYJ is a professor in the Department of Electrophysics, National Chiao Tung University, Hsinchu 300, Taiwan.
